# Studying CaMKII: Tools and standards

**DOI:** 10.1016/j.celrep.2024.113982

**Published:** 2024-03-21

**Authors:** Carolyn Nicole Brown, Karl Ulrich Bayer

**Affiliations:** 1 Department of Pharmacology, University of Colorado Anschutz Medical Campus, Aurora, CO 80045, USA

## Abstract

The Ca^2+^/calmodulin (CaM)-dependent protein kinase II (CaMKII) is a ubiquitous mediator of cellular Ca^2+^ signals with both enzymatic and structural functions. Here, we briefly introduce the complex regulation of CaMKII and then provide a comprehensive overview of the expanding toolbox to study CaMKII. Beyond a variety of distinct mutants, these tools now include optical methods for measurement and manipulation, with the latter including light-induced inhibition, stimulation, and sequestration. Perhaps most importantly, there are now three mechanistically distinct classes of specific CaMKII inhibitors, and their combined use enables the interrogation of CaMKII functions in a manner that is powerful and sophisticated yet also accessible. This review aims to provide guidelines for the interpretation of the results obtained with these tools, with careful consideration of their direct and indirect effects.

## INTRODUCTION

The Ca^2+^/calmodulin(CaM)-dependent protein kinase II (CaMKII) is most famous for its prominent roles in neurons^1,2^ and in the heart.^3,4^ However, at least one CaMKII isozyme is expressed in any cell type examined. Additionally, CaMKII is a multifunctional protein kinase that can phosphorylate a large variety of substrate proteins at Ser/Thr residues and thereby contribute to a variety of Ca^2+^-induced functions. This review aims to help you, the reader, to study the role of CaMKII in your favorite cell type and/or your favorite cellular function. Thus, while we will provide an overview of some sophisticated methods such as light-induced manipulations, we will focus on approaches that are readily accessible: pharmacological inhibitors. Such CaMKII inhibitors now include three different classes with distinct mechanisms of inhibition, making these pharmacological approaches more powerful than ever, especially with some guidance regarding the interpretation of the results. We will emphasize both primary and secondary effects, both for pharmacological inhibitors and for tool mutants. First, however, we will provide a brief overview of the complex regulation of CaMKII.

## THE MOLECULAR BASIS OF CaMKII REGULATION

CaMKII is a family of four isozymes (α, β, γ, and δ) encoded by different genes, with alternative splicing giving rise to more diversity.^1,2,5^ Unless noted otherwise, any amino acid residues mentioned in this review will refer to the brain-specific CaMKIIα isozyme, which was also the first described member of the larger family of CaM kinases^1^; the numbers of the homologous residue in the other three isozymes are typically one numerical value higher.

### CaMKII structure and stimulation by Ca^2+^/CaM

**Figure 1.**
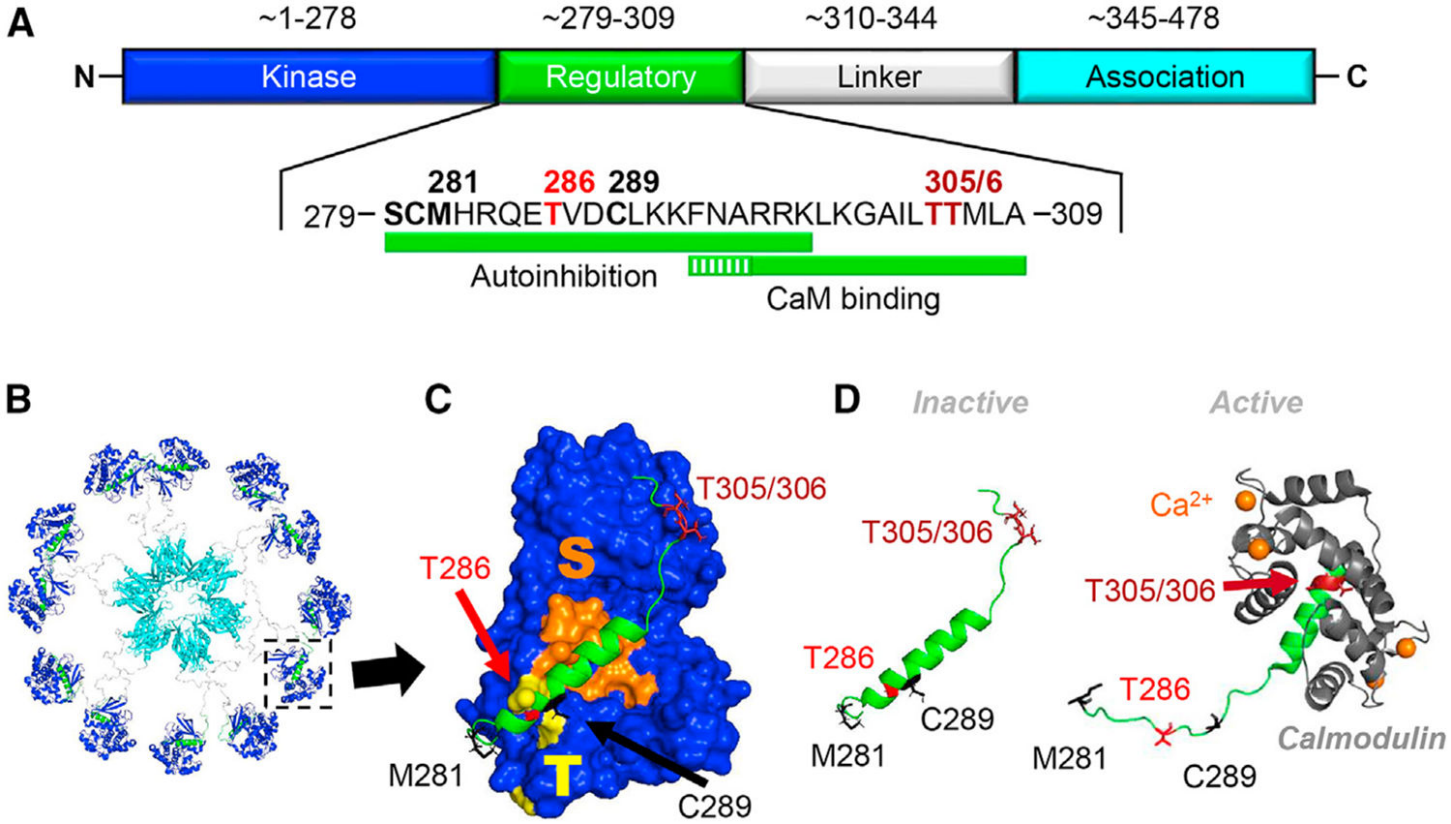
CaMKII structure and regulation (A) All CaMKII isozymes are comprised of a kinase domain, a regulatory domain, a linker region that is subject to alternative splicing, and an association domain. Amino acid ranges and positions are indicated for the major variant of the α isozyme. The kinase domain of the β, δ, and γ contains one more amino acid (inserted after the third position compared to the α isozyme). The linker region of the major β splice variant in brain is 98 amino acids and can be up to 222 amino acids in the β_M_ splice variant. Several sites in the regulatory domain (in bold) can be post-translationally modified to regulate CaMKII activity. The core CaM-binding region is indicated by a solid green bar, while the residues implicated in CaM trapping are indicated by a striped bar. (B) Twelve CaMKII subunits self-associate via the C-terminal association domain to form holoenzymes (PDB: 5u6y). (C) Structure of the CaMKII kinase (surface; CaMKIIα amino acids [aa] 7–274, PDB: 6vzk) and regulatory (ribbon; CaMKIIα aa 281–309, PDB: 5u6y) domains. The S site (substrate-binding site) and T site (T286 docking site) form a continuous groove that under basal conditions is blocked by the regulatory domain. (D) Ca^2+^/CaM binding displaces the regulatory domain to allow access to the S and T sites and to T286 (see also C). Note that this Ca^2+^/CaM binding changes which region of the regulatory domain is in a helical conformation (inactive, CaMKIIα aa 281–309, PDB: 5u6y; active, homologous residues in CaMKIIδ, PDB: 2wel).

Every CaMKII isozyme contains an N-terminal kinase domain, followed by a short regulatory domain that contains the binding site for Ca^2+^/CaM, a variable linker region that is subject to alternative splicing, and a C-terminal association or hub domain (Figure 1A) that mediates the formation of predominantly 12meric holoenzymes (Figure 1B). The holoenzymes are largely in an extended conformation, with the kinase domains radiating outward from the central hub and with the Ca^2+^/CaM-binding site of the regulatory domain accessible^6^ (Figures 1B and 1C). Notably, while the central hub is rigid, the positioning of the kinase domain is much more flexible.^6,7^

The activity of each individual CaMKII subunit is stimulated by the direct binding of Ca^2+^/CaM to its regulatory domain (Figures 1C and 1D). This Ca^2+^/CaM binding likely enhances ATP affinity,^8^ but the vast majority of the dramatic ~1,000-fold increase in enzymatic kinase activity is mediated by enabling substrate access. In the basal state (Figure 1C) the regulatory domain is bound to the kinase domain, in part via interactions of the region around T286 on the regulatory domain with the T286-binding site on the kinase domain (T site), which results also in the block of substrate access to the neighboring substrate-binding site (S site). Ca^2+^/CaM binding to the regulatory domain then displaces it, now allowing substrate access to the S site on the kinase domain, thereby allowing enzymatic kinase activity (i.e., the phosphorylation of the hydroxyl group of a Ser or Thr residue on a substrate protein).

### Ca^2+^/CaM-induced autophosphorylation at T286

Displacing the regulatory domain allows access to several features of CaMKII: (1) to the S site, which enables substrate binding and thereby enzymatic activity (as described above); (2) to the T site, which enables binding to other proteins (as described in the next paragraph); and (3) to T286 on the regulatory domain, thereby making T286 accessible for phosphorylation by a neighboring subunit within the holoenzyme (Figure 1B). Once phosphorylated, pT286 prevents complete reassociation of the regulatory domain with the T site, thereby keeping the CaMKII in a state that is partially active (~20%–40% of maximal activity)^9,10^ independent of the initial Ca^2+^/CaM stimulus. This Ca^2+^-independent state is also termed the “autonomous” state. Importantly, generation of pT286 requires binding of two Ca^2+^/CaM molecules to two neighboring subunits: binding of one Ca^2+^/CaM to one subunit to stimulate kinase activity, and binding of another Ca^2+^/CaM to make T286 accessible for phosphorylation on the other subunit.^11,12^ This second, substrate-directed requirement for Ca^2+^/CaM prevents any T286 phosphorylation in absence of a Ca^2+^ signal and thereby also the “self-perpetuation” of the pT286 state that had been proposed before this dual requirement for CaM was known.

### Ca^2+^/CaM-induced GluN2B-like protein binding

Interactions with other proteins can occur on many CaMKII surfaces,^13,14^ but interactions with the S site or T site are of special interest, as they can be regulated by Ca^2+^/CaM and by pT286.^15^ The S and T sites form a continuous groove on the CaMKII kinase domain and are proposed to act in concert for protein binding.^16,17^ For instance, the Ca^2+^/CaM-induced binding of CaMKII to the NMDA-type glutamate receptor subunit GluN2B is proposed to progress from an initial S-site binding mode to a more stable T-site binding mode that no longer requires the initial Ca^2+^/CaM stimulus.^15,16^ The main CaMKII binding site on GluN2B is located around S1303 and has homology to the CaMKII regulatory region around T286. This T286 region of CaMKII exemplifies (1) S-site binding by its interaction during its phosphorylation by a neighboring subunit and (2) T-site binding by its interaction in the inactive basal state (see Figure 1C). Notably, similar to pT286, GluN2B binding is proposed to keep the regulatory domain displaced and indeed induces Ca^2+^-independent autonomous CaMKII activity.^15,16^ The only available crystal structure shows GluN2B in the S-site binding mode,^17^ as this study used a shorter GluN2B-derived peptide that appears to favor S- over T-site binding.^16^ Thus, the precise nature of the GluN2B binding mode that supports autonomous CaMKII activity still awaits final validation.

Several other proteins have been suggested to bind CaMKII in a mode similar to that of GluN2B, including Tiam1,^18^ connexin-36,^19^ KIF17,^20^ densin-180,^21^ and several voltage-dependent Ca^2+^ channels (VDCCs).^22-24^ The regulation of CaMKII binding is understood to be best for GluN2B. As expected, this type of binding is generally positively regulated by Ca^2+^/CaM and by pT286 (with absolute requirement for at least one of these two stimuli) and negatively regulated by phosphorylation of GluN2B at S1303 within the binding region. More unexpectedly, the binding also has strong dependence on the occupation state of the ATP-binding pocket on CaMKII,^21,24,25^ which can be fulfilled by binding of either ATP, ADP, or–as discussed later–even various ATP-competitive kinase inhibitors, at least in the case of GluN2B binding.^25-27^

### Other “autonomy” mechanisms also require an initial Ca^2+^/CaM stimulus

As reviewed previously,^1,3,4^ autonomous CaMKII activity can be induced not only by pT286 or GluN2B binding but also by GlcNAcylation at S279, oxidation at C280/M281/C289, or *S*-nitrosylation at C280/C289 within the CaMKII regulatory region^28-30^ (see Figures 1C and 1D). Notably, only CaMKIIα contains C280, whereas all other isozymes contain a methionine residue in the homologous position, which can only be oxidized but not *S*-nitrosylated. The cellular functions of these mechanisms are generally not yet well understood, but hypo-nitrosylation of CaMKIIα impairs learning, likely through a chronic effect on synaptic CaMKII localization.^31^ CaMKII oxidation and GlcNAcylation both contribute to pathologies in the heart.^3,4^ Notably, all forms of autonomous CaMKII have in common that their generation appears to require Ca^2+^/CaM, likely for making the residues accessible for the respective modifications, as indeed well demonstrated for pT286. Thus, even though these mechanisms lead to Ca^2+^-independent kinase activity, they all require an initial Ca^2+^ stimulus.

### Inhibitory autophosphorylation at T305/306 and crosstalk of regulation

In contrast to pT286, a nearby autophosphorylation at T305/306 (pT305/306) is inhibitory and can occur both within the same subunit^8,32^ as well as between neighboring subunits.^33^ The T305/306 residues are within the Ca^2+^/CaM-binding region of the autoregulatory domain (Figures 1C and 1D), and their phosphorylation is mutually exclusive with Ca^2+^/CaM binding. Additionally, pT305/306 directly reduces GluN2B binding and the autonomous activity of pT286 CaMKII.^33,34^ After dissociation of CaM, a kinase subunit with pT286 can phosphorylate itself at pT305/306; this subunit is then still autonomously active (which is ~20%–40% of maximal activity),^9^ but subsequent stimulation by Ca^2+^/CaM to the maximal activity level is prevented. Additionally, a kinase subunit with pT286 can also induce pT305/306 on a neighboring subunit without pT286 as long as this neighboring subunit does not have Ca^2+^/CaM bound to it^33^; notably, this can completely shut down this neighboring subunit, as pT305/306 then prevents both future stimulation and pT286. In neurons, the regulatory crosstalk between Ca^2+^/CaM, pT286, pT305/306, and GluN2B binding appears to be crucial for signal computation that leads to strengthening versus weakening of synaptic connections.^33^ The complex regulation of CaMKII is likely to contribute to similar computation of Ca^2+^ signals also in other non-neuronal systems; however, this still awaits thorough investigation.

### Holoenzyme considerations

The holoenzyme significantly shapes CaMKII regulation, especially pT286, pT305/305, and their crosstalk. Even though pT286 does not strictly require the holoenzyme,^11,35^ the reaction that occurs within the holoenzyme is likely more physiologically relevant than the reaction between holoenzymes, as the former reaction is much faster.^36^ Additional interactions within the holoenzyme may further tune CaMKII regulation: While most kinase subunits within a holoenzyme are in an extended conformation,^6,7^ some fraction of kinase domains may fold back to the association domain in a compact conformation that restricts Ca^2+^/CaM access.^6,37,38^ Additionally, some kinase subunits within the holoenzyme may interact via their regulatory domains, which would also restrict Ca^2+^/CaM access and make activation cooperative.^7,39,40^ Finally, even though the CaMKII holoenzyme is very stable, a dynamic subunit exchange has been reported.^41,42^ However, such exchange has been questioned^35^ and possible functions remain speculative, although repair of holoenzymes with damaged subunits would be one obvious possibility.

## THREE DISTINCT CLASSES OF CaMKII INHIBITORS: A POWERFUL COMBINATION

To determine whether or not CaMKII is involved in any specific cellular function, it is best to start with pharmacological inhibitors. The availability of three classes of CaMKII inhibitors has now made this approach more powerful (Table 1 and Figure S1), especially when the results are carefully interpreted in light of the specific distinct inhibitory mechanisms.

### Ca^2+^/CaM-competitive inhibitors

The traditional CaMKII inhibitors KN62 and KN93 are competitive with activation by Ca^2+^/CaM.^43,44^ Thus, they inhibit the Ca^2+^/CaM-induced stimulation of both enzymatic activity and protein-protein interactions, such as the binding to GluN2B.^50^ Additionally, they are very effective at blocking the generation of autonomous activity by pT286. However, once autonomous activity is generated (by pT286 or another mechanism), these inhibitors do not interfere with this autonomous CaMKII activity,^50^ as it is no longer dependent on Ca^2+^/CaM. Although KN inhibitors are relatively selective inhibitors,^51^ they also block CaMKIV^52^ and synaptic protein kinase C (PKC).^53^ Additionally, they directly inhibit VDCCs and voltage-dependent K channels.^54-57^ It is somewhat surprising that KN93 does not inhibit more CaM kinases, as its inhibitory mechanism appears to involve direct binding to CaM.^58,59^ However, the inhibition of synaptic PKC is related to this CaM effect^53^: KN62 and KN93 can directly induce CaM binding to AKAP79/150 (a synaptic anchoring protein for PKA, PKC, and calcineurin^60^) and thereby displace PKC from its binding site. A CaM-binding site also appears to be required for inhibition of the IP3 receptor type 1 (IP3R1) by KN93.^61^ The mechanism of VDCC inhibition is less clear, but this side effect significantly compromises the utility for studying CaMKII functions that rely on VDCCs for the upstream Ca^2+^ signal.

### T-site binding peptide inhibitors

All current peptide inhibitors of CaMKII can bind to the groove that contains the neighboring S and T sites (see Figure 1C). The S-site binding helps inhibit enzymatic activity by blocking substrate access; the T-site binding additionally inhibits GluN2B-like binding to other proteins. Peptide inhibitors such as AC3-I or autocamtide-2-related inhibitory peptide (AIP) were designed based on the CaMKII regulatory region around T286,^62-65^ which binds to the T site.^15^ Other peptide inhibitors such as CaM-KIINtide, tatCN21, or tatCN19o were designed based on the natural endogenous CaMKII inhibitor protein CaM-KIIN^66^; they are highly selective for CaMKII and also bind to the T site.^45,67^ The inhibitory region of these latter peptides was determined to be contained within a 19meric peptide, which was then further optimized to enhance potency and selectivity.^46^ According to the mechanism of action of the peptides, they block both Ca^2+^/CaM-stimulated and autonomous CaMKII activity equally well.^47,50,68^ The exception may be autonomous activity that is generated by GluN2B-like protein binding via the T site, as such binding could interfere with the access of the peptides to the bound CaMKII. However, while it is clear that the peptide inhibitors can prevent GluN2B binding^16,45^ and related protein interactions,^18,22,23^ the effects on activity of already GluN2B-bound CaMKII remains to be tested experimentally, as the additional S-site interactions of the T-site binding peptides may still allow for inhibition.

Importantly, the peptides have successfully been made cell-penetrating by fusion with the tat sequence^45,68^ or by myristoylation^69^; the tat sequence also conferred blood-brain-barrier penetration for *in vivo* applications.^47^ The tatCN19o peptide is the most potent one of these inhibitors^47^; however, tatCN21 has been used more widely and is thus better established.

### ATP-competitive inhibitors

The third class is the newest: CaMKII-specific ATP-competitive inhibitors such as AS105, AS283, or AS397 (developed by Allosteros).^26,49,70^ Similar to older broad-spectrum ATP-competitive inhibitors such as staurosporine or H7, these compounds block enzymatic CaMKII activity but not GluN2B binding.^26,27^ In fact, they can even enhance GluN2B binding, likely by eliminating the negative-regulatory phosphorylation of GluN2B at S1303 by CaMKII (which overall appears to outweigh the additional elimination of the positive regulation of the binding by pT286).^26^ They also block autonomous activity generated by pT286, GluN2B binding, or other mechanisms.

Unfortunately, the Allosteros inhibitors are not yet commercially available, although several other inhibitors are. For instance, ruxolitinib, a clinically used JNK (inhibitor of c-Jun N-terminal kinase), is also an excellent ATP-competitive CaMKII inhibitor.^49^ GS-680 (developed by Gilead) is a selective and potent CaMKII inhibitor, albeit with a ~7-fold preference for the δ isozyme over the brain-specific α isozyme.^71^ Some other options that may be less potent and/or selective include rimacalib (SMP-114),^72^ 3′,4′-dihydroxyflavonol (DiOHF; NP202),^73,74^ and the src kinase inhibitor bosutinib.^38^ RA608 (developed by Sanofi) appears to be CaMKII selective and has a similar preference for the δ isozyme as GS-680, but is overall less potent and has an especially low inhibitory potency for the β isozyme,^75^ which could be an advantage for some specialized applications.

### Non-inhibitor compounds that affect the association domain

Two distinct classes of compounds that act on the association domain are worth mentioning alongside the three classes of CaMKII inhibitors. One peptide derived from the CaMKII regulatory region around the T305/306 autophosphorylation sites (see Figure 1) has been described to disrupt holoenzymes.^76^ So far, this tool has only been used *in vitro*, but it will be interesting to see a similar holoenzyme disruption within cells. The other compounds are analogs of γ-hydroxybutyrate (GHB) that have been described to stabilize the holoenzyme.^77^ Whereas GHB can additionally bind to GABA receptors, analogs such as HOCPCA and Ph-HTBA are more CaMKII selective.^77,78^ Notably, whereas GHB and HOCPCA had no effect on enzymatic CaMKII activity,^77^ Ph-HTBA additionally interfered with pT286 and substrate phosphorylation within cells.^78^ Interestingly, HOCPCA protected neurons from ischemic injury and reduced glutamate-induced co-localization of CaMKII and GluN2B,^77^ but the mechanism of how CaMKII holoenzyme stabilization leads to neuroprotection remains largely speculative at this point.

### Controls and interpretations of results obtained with the inhibitors

Negative controls can include scrambled or reversed sequences for the peptide inhibitors or the inactive KN92 for the KN93 inhibitor. However, KN92 does not control for the inhibition of CaMKIV or PKC, and it is unclear whether it inhibits VDCCs equally as well as KN93. Thus, a more effective approach is using positive controls instead, specifically two or three different inhibitors with distinct mechanisms (Table 1). Any individual inhibitor is likely to have some off-target effect, but these effects likely differ among the inhibitors. Thus, an effect by two distinct inhibitors strongly indicates CaMKII involvement; an effect by all three inhibitor classes makes CaMKII involvement almost certain. Additionally, this multiple-inhibitor approach can distinguish between stimulated and autonomous CaMKII activity and even has the potential to distinguish between enzymatic and structural functions: Generation of autonomous activity can be blocked by all inhibitors, but CaM-competitive inhibitors do not affect autonomous activity once it is generated. Structural functions mediated by GluN2B-like binding can be prevented by both CaM-competitive and T-site peptide inhibitors, but only T-site peptide inhibitors have the potential to disrupt the binding even after it has formed, at least at higher concentrations.^79^ By contrast, ATP-competitive inhibitors do not interfere with GluN2B-like binding; if anything, they may enhance it.^26^

In the case of a specific cellular function being affected by only one of three CaMKII inhibitors, the most likely interpretation is that CaMKII is not involved. It would then be advisable to test involvement of some of the known off-target effects of the inhibitor, which can readily be done pharmacologically in most cases. For KN93, this would include inhibition of VDCCs, synaptic PKC, or CaMKIV (for instance by nimodipine, Go6983, or STO-609, with the latter inhibiting CaMKIV indirectly); for ruxolitinib, it would include inhibition of JAK1/2 (for instance by tofacitinib). However, in exceptional cases, it is possible that certain CaMKII functions are indeed only blocked by one of the three inhibitor classes. For instance, it is possible that the autonomous activity of a CaMKII in GluN2B-like binding mode is inhibited only by the ATP-competitive inhibitor but neither by the CaM-competitive inhibitor nor by the T-site binding peptide inhibitor (although the latter has not yet been formally tested for any of the peptide inhibitors).

Another slightly more obscure potential consideration in the case of an unusual result is that some of the inhibitors could actually enhance certain CaMKII functions depending on their specific mechanism of action. For instance, CaMKII binding to GluN2B has been described to increase binding of the CaMKII regulatory domain to α-actinin-2,^79^ and the peptide inhibitors could potentially have a similar effect, as they appear to bind in a way similar to that of GluN2B. Similarly, at least some ATP-competitive CaMKII inhibitors appear to directly enhance the Ca^2+^/CaM-induced CaMKII binding to GluN2B.^28^

### Some practical considerations

For use in cell culture or slice preparation, good starting concentrations for some of the CaMKII inhibitors are 10 μM KN93, 5 μM tatCN21, and 10 μM AS283 (Table 1). For the alternative ATP-competitive inhibitors AS397, ruxolitinib, or GS-680, a similar concentration should suffice. For the much more potent tatCN19o peptide, a minimum starting concentration of 2 μM should be used. For other peptides, a higher starting concentration would be preferable.

The estimates above are based on the potency and on past successful use of these inhibitors. However, such extrapolation has to be taken with a grain of salt, as the equivalent doses can differ between different experimental preparations. For instance, although the myrCN27 peptide (also termed myr-CaM-KIINtide) showed effects even at 1 μM, this may have been specific to a membrane-proximal function of CaMKII (where myrCN27 may enrich) and may not be the same for cytosolic functions. Additionally, some cellular functions might be dramatically impaired by reducing CaMKII activity by half, whereas other cellular functions may completely depend on CaMKII activity but may be sufficiently mediated by 5% of this activity. There also may be other dose-dependent inhibitor effects. For instance, 5 μM tatCN21 was sufficient to prevent CaMKII interaction with GluN2B that is required for long-term potentiation induction in hippocampal slices^26,68^; however, 20 μM tatCN21 appeared to be necessary to disrupt this interaction after its formation.^79^

When basing the starting concentration of a drug on its potency, a good rule of thumb for blocking most of the activity is ~10× the half-maximal inhibitory concentration (IC_50_) in biochemical assays and ~50–1,000× IC_50_ in pharmacological experiments in cells or tissues. However, for CaM- or ATP-competitive inhibitors, it is important to know which CaM or ATP concentrations were used to determine the IC_50_. For instance, biochemical CaM kinase assays typically use 0.3–1 μM CaM and 50–100 μM ATP, whereas cells typically contain 3–10 μM CaM and ~4 mM ATP. To make matters worse, the IC_50_ data available for many drugs do not indicate the assay conditions used. Thus, *K*_i_ values are typically preferable, as they do not rely on the assay conditions. Two equations are listed below to help convert *K*_i_ to IC_50_ (and vice versa) at specific CaM or ATP concentration (for instance the expected cellular concentrations) for competitive inhibitors. The formula requires knowledge of the half-maximal effective concentration (EC_50_) for CaM or *K*_M_ for ATP. A good value for the CaM EC_50_ is 30 nM^41,42^ (although this may be as high as 100 nM for the α isozyme and as low as 15 nM for other isozymes)^37,38,40^ and 30 μM for the *K*_M_ for ATP^26^ (with reported values ranging from 8 to 125 μM).^8,70,80,81^

For CaM-competitive inhibitors: IC_50_ = *K*_i_ (1 + [CaM]/EC_50_ for CaM).

For ATP-competitive inhibitors: IC_50_ = *K*_i_ (1 + [ATP]/*K*_M_ for ATP).

Another consideration when using a drug is its solubility and stability. While most of the peptide inhibitors are directly water soluble, the stock solutions for most of the other inhibitors should be made up in DMSO (see Table 1). After preparation of stock solutions, best practice is to freeze them in aliquots to avoid unnecessary freeze-thawing. Light exposure should be minimized but should not be a concern for most CaMKII inhibitors (in contrast to some VDCC inhibitors that are extremely light sensitive).

## MUTATIONS OLD AND NEW: UNDERSTANDING THEIR EFFECTS

Specific mutants have long been used to better understand specific aspects of CaMKII regulation and function (Table 2). This has included simple overexpression of mutants, knockdown/re-expression approaches, and generation of constitutive or inducible mutations in mice. To allow better interpretation of the results, we discuss here several specific mutations for both their primary and secondary effects.

### Phospho-site and regulation mutations

The alanine or valine mutation of T286, T305/306, C280, M281, or C289 prevents their phosphorylation, oxidation, or *S*-nitrosylation, respectively, and thereby ablates the regulatory functions of these modifications. To mimic phosphorylation, aspartate or glutamate mutations have been used. Indeed, a T286D mutant is autonomously active, and a T305/306D mutant cannot bind to Ca^2+^/CaM. However, it should be noted that aspartate or glutamate residues are much less charged and bulky than phosphorylated serine or threonine residues; thus, these mutations can for some functions mimic the non-phosphorylated state instead of the phosphorylated state.

Consistent with the crosstalk of regulation, these CaMKII mutants also have secondary effects. For instance, a T286A mutant can indirectly also reduce pT305/306 and GluN2B-like binding.^25,33,87^ A T286D mutant can directly promote pT305/306. As such, a T286D mutant behaves similarly to T286/305/306D triple mutant, with both having the opposite effect on synaptic plasticity as a T286D/305/306A mutant that mimics pT286 without additional pT305/306.^34,88^ Notably, without additional T286D mutation, a T305/306D mutant is completely inactive (see next subsection). As such, for studying the function of regulation by this phosphorylation, ablating the phosphorylation by alanine or valine mutations is more useful. Similarly, for pT286, the T286D mutation uncouples generation of autonomy from the input signal, which can impair a cellular function just as much as ablating this form of autonomy.

### Mutants to ablate enzymatic activity: Dominant negative?

The mutants most commonly used to ablate enzymatic CaMKII activity have been K42M and K42R. Both mutants act by disturbing the ATP-binding pocket, which disrupts both enzymatic activity and GluN2B binding (as nucleotide binding to the kinase domain is required not only for enzymatic activity but also for efficient GluN2B binding^25-27,82^). Expression of these kinase-dead mutants has been used to interfere with endogenous CaMKII signaling to elicit a dominant-negative effect. The mechanisms of such dominant-negative effects are unclear but could occur through interference with pT286, GluN2B-like binding, or substrate binding. Importantly, even though a dominant-negative effect of these mutants has been shown for some cellular functions,^89-91^ a similar dominant-negative effect on other cellular CaMKII functions should not be assumed a priori.

CaMKII activity can also be ablated by mutations that prevent Ca^2+^/CaM binding. These mutations include T305/306D (see previous subsection) and L299/I303/304Q,^34^ which was designed to prevent Ca^2+^/CaM binding without introducing negative charges. Additionally, these mutants abolish GluN2B-like binding of CaMKII. Thus, similar to the K42 mutations, they may elicit some dominant-negative effects and cannot be used to distinguish between enzymatic and structural functions of CaMKII.

Recently, the D135N mutation has been suggested to ablate enzymatic activity without affecting GluN2B-like binding.^84^ The D135 residue is homologous to D166 in PKA, which interacts with the hydroxyl group of the substrate residue phosphorylated during a kinase reaction.^92,93^ Thus, the CaMKII D135N mutant may provide a very useful new tool, although it awaits more extensive characterization regarding remaining levels of both enzymatic activity and GluN2B binding. Notably, the D135N mutation appears to require an additional T286D mutation in order to support cellular functions that depend on GluN2B binding; this would be consistent with suppression of pT286 by this mutation, which in turn would reduce GluN2B binding.

### Mutants to ablate protein interactions

GluN2B binding can be ablated by various mutations, but several of them also ablate enzymatic activity (such as K42M or T305/306D; see previous subsection). By contrast, two mutations within the CaMKII T site impair GluN2B binding but retain significant enzymatic activity^94^: I205K and W237K. The W237K mutation additionally directly generates significant autonomous kinase activity^94^ in addition to interfering with GluN2B binding slightly less than I205K.^16^ Thus, the I205K mutant is used more commonly. However, the I205K mutant is also overlapping with the S site and is likely to have at least some effect also on substrate binding, which may differ between specific substrates.^9,17,94^ Additionally, as expected from disrupting T-site binding, the mutation also disrupts binding to several other proteins, including Tiam1,^18^ densin-180,^21^ at least one VDCC,^24^ and likely others. Thus, I205K remains a very useful tool, but the function of specific protein-protein interactions should be followed up also by mutations on the other binding partner. For GluN2B, this includes the L1298A/R1300Q mutation.^95-97^

To ablate the F-actin binding that is prevalent in the CaMKIIβ isozyme, the naturally occurring splice variant βe can be used,^98,99^ which deletes a small exon coding for amino acids 316–340; however, this deletion also appears to reduce the CaMKIIβ affinity for Ca^2+^/CaM, approximately to the levels normally seen in the α isozyme.^100^ Similarly, alternative splicing can include or exclude a nuclear localization signal that interacts with the nuclear import machinery. This signal is included in the minor splice variant αB and can be present also in the other isozymes except for β, which lacks the corresponding intron completely.^101^

### Pharmacogenetics: The “Shokat mutant” and beyond

The “Shokat mutation” (F89G in CaMKII) is designed to enlarge the ATP-binding pocket of a kinase to allow access of the inhibitor NM-PP1, which does not affect the activity of wild-type kinases.^102^ This pharmacogenetic approach allows for exquisite selectivity and controls. Surprisingly, in CaMKII, the F89G mutation not only allowed access of NM-PP1 but also dramatically reduced ATP binding.^26^ As such, the F89G mutant acts much as the K42M mutant: it inhibits not only enzymatic activity but also GluN2B binding.^26^ However, addition of NM-PP1 rescues GluN2B binding (while further suppressing enzymatic activity). Like other ATP-competitive CaMKII inhibitors, it also makes the GluN2B binding in cells independent of pT286.^26^ Thus, in contrast to the D135N mutation (see two subsections above), the F89G mutant does not require additional T286D mutation to mediate cellular functions and can be used in pharmacogenetic combination approaches, i.e., any effect that requires GluN2B binding should be seen with the F89G only after rescue with NM-PP1. Notably, similar pharmacogenetic effects were achieved using a CaMKII T286A mutant in combination with a regular ATP-competitive inhibitor.^26^ Similar effects for other GluN2B-like interaction partners remain to be tested but would be expected if they strongly depend on nucleotide binding to CaMKII.

## READOUTS FOR CaMKII ACTIVITY AND LOCALIZATION IN CELLS AND TISSUE

A variety of live-imaging approaches have been developed to assess CaMKII activation and movement within cells. They have several advantages and certainly provide a good complement to more traditional approaches.

### The pT286 readout is not equivalent to activity

CaMKII activity^9,40^ (or CaMKII binding to GluN2B)^25-27^ can be measured in controlled biochemical assays that are well suited to study the mechanisms of regulation. However, these assays are ill suited for measuring CaMKII activity in cells or tissue: while cells or tissues can be homogenized for use in these biochemical assays, the level of activity measured will depend more on the inclusion or exclusion of Ca^2+^ in the assay buffer and less on the endogenous CaMKII activation state. Thus, a common alternative approach has been to instead measure the level pT286 in homogenates by western blot. Indeed, measuring the pT286 level provides useful and relevant information. However, it does not provide a readout of the acute Ca^2+^/CaM-stimulated CaMKII activity. Additionally, it is not clear how long pT286 will last after a Ca^2+^ stimulus, as this will depend on the activity of phosphatases; in either case, phosphatase inhibitors need to be added during the homogenization in order to prevent dephosphorylation during preparations of the extracts.

### Three classes of optical sensors to assess CaMKII activation

**Figure 2.**
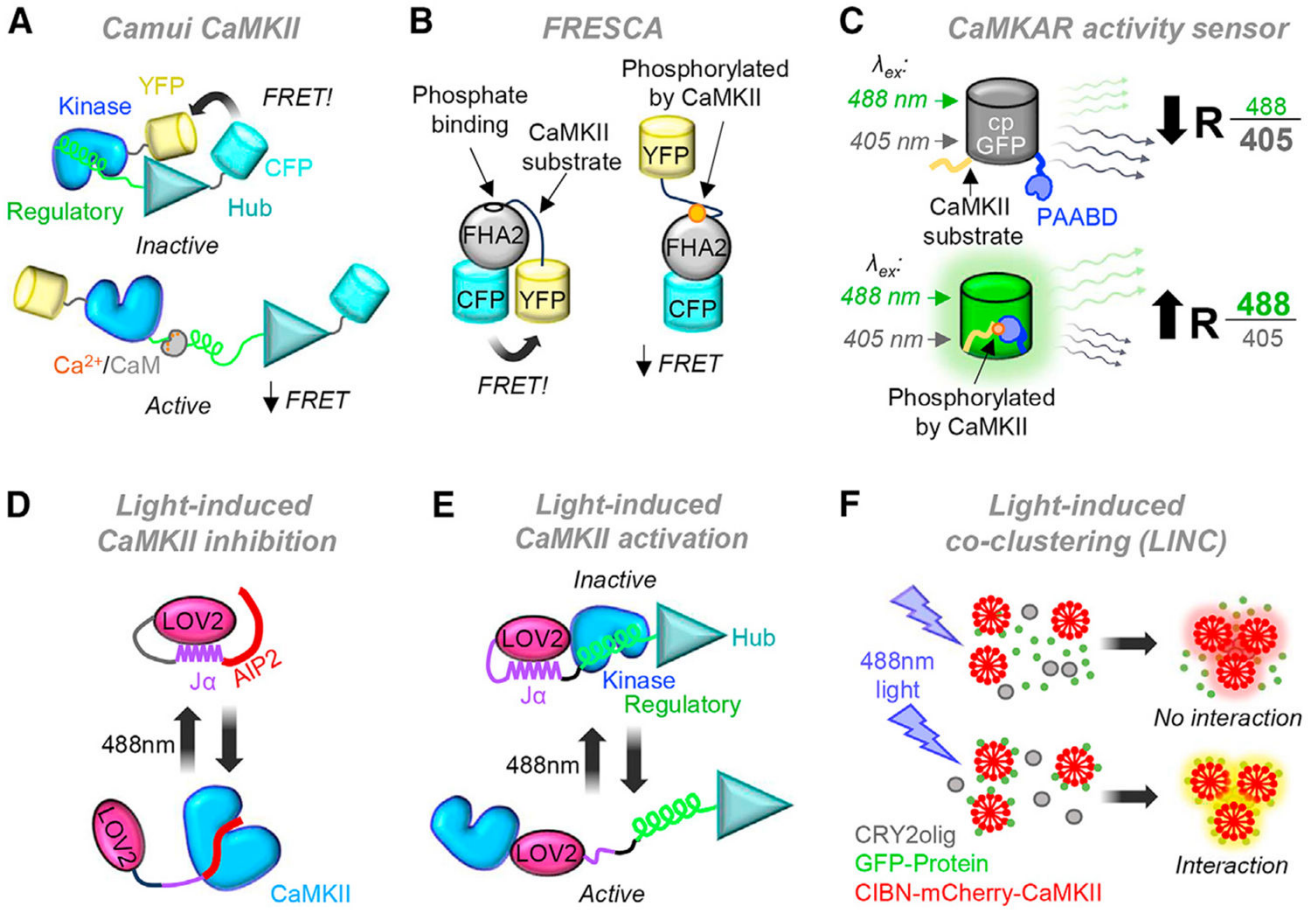
Optogenetic tools for monitoring and controlling CaMKII activation (A) Camui is an FRET-based sensor that detects conformational changes in CaMKII upon activation. An N-terminal YFP and C-terminal CFP undergo FRET while CaMKII is in the inactive state. A conformational change occurs upon activation by Ca^2+^/CaM, resulting in decreased FRET. Camui is illustrated here as a monomer but is thought to form holoenzymes. (B) FRESCA is an FRET-based sensor of CaMKII activity that uses a modified syntide2 peptide substrate. Under basal conditions, the N-terminal CFP and C-terminal YFP undergo FRET. Upon CaMKII activation and phosphorylation of the syntide2-derived region, the phosphorylated residue binds FHA2, causing a conformational change and decreased FRET. (C) CaMKAR is a CaMKII activity reporter that consists of an N-terminal CaMKII substrate (MHRQETVDCLK) and a C-terminal phosphorylated amino acid binding domain (PAABD) connected by a circularly permuted GFP (cpGFP). This reporter is excitation ratiometric, as emission by 488-nm excitation increases upon phosphorylation of the substrate by CaMKII (and phosphorylation of the substrate), while emission by 405-nm excitation decreases or remains the same. (D) paAIP2 consists of an N-terminal LOV2-Jα domain fused to a C-terminal CaMKII-inhibitory peptide (an improved AIP-related peptide inhibitor) to generate paAIP2. Activation with 488-nm light causes undocking and unwinding of the Jα helix, resulting in exposure of the AIP2 peptide for CaMKII inhibition. (E) paCaMKII contains the light-sensitive LOV2-Jα domain inserted between the CaMKII kinase and regulatory domains. Activation with 488-nm light causes undocking and unwinding of the Jα helix, activating CaMKII, similarly as normally achieved by Ca^2+^/CaM stimulation. Note that paCaMKII can still also be stimulated by Ca^2+^/CaM, and that either light or Ca^2+^/CaM makes its T286 homolog accessible for autophosphorylation. The paCaMKII is illustrated here as a monomer but is likely to form dimers but not holoenzymes. (F) Light-induced clustering of CaMKII is achieved by expression and optical manipulation of three constructs: CRY2olig, CIBN-mCherry-CaMKII, and a GFP-tagged protein of interest. Activation with 488-nm light causes both CRY2olig oligomerization (tetramerization) and association of CRY2olig with CIBN, resulting in the formation of red puncta. If CaMKII interacts with the GFP-tagged protein of interest, this protein will co-cluster with the others, resulting in yellow puncta.

The first two classes of optical CaMKII activation sensors are fluorescence resonance energy transfer (FRET) based and can be adapted for use in fluorescence lifetime imaging (FLIM) and with different pairs of fluorescent proteins. The Camui class of sensors uses fusion of two fluorescent proteins to the N and C termini of CaMKII.^103,104^ Ca^2+^/CaM binding to the sensor then causes a structural change that results in a change in FRET or FLIM^103,104^ (Figure 2A). Somewhat surprisingly, the C-terminal fusion does not appear to prevent holoenzyme formation of the Camui sensors, and the Camui sensors can undergo autophosphorylation at T286.^103^ Although Camui senses structural changes rather than actual activity, it appears that the relative readout for full activation by Ca^2+^/CaM binding (i.e., maximal activity) versus pT286-induced autonomy (i.e., 20%–40% of maximal activity) by biochemical CaMKII assays and by Camui are very similar.^9,105^

Two newer sensors are instead based on actual substrate phosphorylation. One is termed FRESCA, for FRET-based sensor of CaMKII activity.^106^ It is based on the C kinase activity reporter sensor for PKC activity^107^ but instead contains a CaMKII substrate region (modified on the basis of the syntide2 peptide). Upon phosphorylation, the phosphate-binding protein (FHA2) domain of the sensor binds the substrate region to cause a conformational change in the sensor that reduces FRET (Figure 2B). The other phosphorylation-based sensor is termed CaMKAR, for CaMKII activity reporter,^49^ and is based on ExRai-AKAR2, a reporter of PKA activity.^108^ The sensor also uses binding of a related domain (FHA1) to a phosphorylated substrate region. However, rather than using FRET, this sensor uses restoration of fluorescence in a circularly permutated GFP (cpGFP), i.e., a single fluorophore (Figure 2C). The CaMKAR reporter has been described to have several advantages over the other reporters, including higher sensitivity.^49^

Notably, CaMKII inhibitors should have different effects on the readout by Camui compared to the newer CaMKII sensors. The signal from the newer phosphorylation-based sensors should be reduced by any CaMKII inhibitor, whereas the signal of the structural Camui sensor may be reduced only by CaM-competitive inhibitors, with no predicted effects by ATP-competitive inhibitor and the potential for enhancement by the peptide inhibitors that may provide a structural wedge. Indeed, ATP-competitive inhibitors have been shown at least to reduce the readout by FRESCA^106^ and CaMKAR^49^ but not by Camui.^49^

### Following CaMKII on the move

In contrast to CaMKII activity, CaMKII localization can be directly assessed in tissue using either immunohistochemistry or biochemical fractionation. However, live imaging of CaMKII localization has many advantages and has been especially powerful in neurons, where CaMKII moves to different synapse types in response to different stimuli. This has typically been done using expression of CaMKII fusion proteins with a fluorescent protein at the N terminus.^109^ As CaMKII is multimer, it is important to use a monomeric fluorescent protein (such as the A207K mutant of EGFP)^110^ to avoid formation of large aggregates.^16^ In neurons, overexpression of labeled CaMKII has typically not posed a problem, likely due to the high expression levels of endogenous CaMKII. However, there may be exceptions, and it may be prudent to use short hairpin RNA against endogenous CaMKII in a knockdown/replacement approach.^34^ Alternatively, for the CaMKIIα isozyme, there is a FinGR intrabody available that enables live imaging of the endogenous protein, and this intrabody can be expressed together with intrabodies against PSD95 and gephyrin, two marker proteins for excitatory versus inhibitory synapses.^33,111,112^ The readouts obtained with the intrabody versus direct GFP fusion have been very similar but with slightly less signal of synaptic enrichment obtained with the intrabody, likely due to some background of intrabody that is not bound to CaMKII.

## SEEING THE LIGHT: OPTICAL MANIPULATIONS OF CaMKII IN SINGLE CELLS

The first optogenetic approaches used channelrhodopsin-2 for light-induced depolarization of individual cells.^113^ These approaches allowed powerful temporal and spatial control of the manipulation but were largely restricted to neuroscience applications. Second-generation optogenetic tools can be applied more generally, as they include light-induced induction or disruption of protein-protein interactions.^114,115^ Here, we describe three such approaches that have been adopted to enable light-induced CaMKII manipulations.

### Light-induced CaMKII inhibition

The light-sensitive LOV2-Jα domain from phototropin1 has been used to engineer tools for photomanipulation of various proteins,^114,116^ including for CaMKII photoactivation^117^ and photoinhibition.^118^ For CaMKII inhibition, the LOV2-Jα domain was fused to the N terminus of an improved AIP-related peptide inhibitor^65^ to generate paAIP2, a photoactivatable peptide inhibitor of CaMKII^118^ (Figure 2D). Blue light triggers rapid undocking and unwinding of C-terminal Jα helix, resulting in exposure of the AIP2 peptide to enable CaMKII inhibition. In the dark, the LOV2-Jα domain spontaneously folds back into a closed conformation, and for paAIP2, loss of CaMKII inhibition occurs over a ~40-s time frame.^118^ This enables precisely controlled inhibition of endogenous CaMKII activity in the cells that express paAIP2.^18,48,118,119^ To monitor expression of paAIP2, it was fused with a fluorescent protein^118^; to facilitate widespread expression, it has also been used in an adeno-associated viral (AAV) vector.^118^ As with any of the T-site binding peptide inhibitors, paAIP2 should inhibit both CaMKII activity and GluN2B-like binding interactions.

### Light-induced CaMKII activation

To create photoactivatable paCaMKII (Figure 2E), the light-sensitive LOV2-Ja domain was inserted between the CaMKII kinase and regulatory domains.^117^ This paCaMKII is readily activatable by either Ca^2+^/CaM or by blue light, which also directly induces both pT286^117^ and GluN2B binding.^26^ The time course of light-induced pT286 appears to closely mimic that of endogenous CaMKII in neurons, with instantaneous induction after stimulation and reversal within ~1 min.^117^ GluN2B binding is also similarly regulated, with normal stimulation-induced GluN2B-mediated synaptic accumulation involving regulation by pT286.^26,87^ For stabilization, paCaMKII contains several mutations in the hub domain,^117,120^ and one of them, F394L, is likely to make paCaMKII dimeric when expressed by itself.^121^ This mutation should still allow pT286 within the dimer as well as binding of the dimer to additional endogenous CaMKII subunits,^121^ as was indeed observed.^117^ As with paAIP2, versions of paCaMKII have been made with various tags and in an AAV vector. In contrast to paAIP2, paCaMKII does not directly affect endogenous CaMKII; an advantage of this is that additional mutations can be introduced into the paCaMKII transgene to study more details of the regulatory mechanism involved in the cellular functions that are induced by the light activation.

### Light-induced CaMKII sequestration

In addition to disrupting interactions, blue light can also be used to induce binding, specifically of the *Arabidopsis* photoreceptor CRY2 (or optimized versions such as CRY2olig) tothe CRY2-binding protein CIB1 (or to a truncated version such as CIBN).^115,122^ This has been used to develop a light-induced co-clustering assay,^123^ which was also successfully adopted to study CaMKII interactions^101,123^ (Figure 2F): when co-expressed with CRY2olig (either with or without a fluorescent label), a CIBN-fused and mCherry-labeled CaMKII (CIBN-mCh-CaMKII) forms clusters within cells after stimulation with blue light. Any co-expressed binding partner of CaMKII (labeled with an appropriate fluorophore) then co-clusters with the CIBN-mCh-CaMKII. Thus, this method can be thought of as a live-imaging version of a co-immunoprecipitation assay, with the added advantage that the observed interaction occurs within the undisturbed cellular milieu. Additionally, as CIBN-CaMKII should also co-associate with endogenous CaMKII, at least after sufficient duration of expression, the method could be used to sequester CaMKII and thereby disrupt its cellular function. Notably, such CaMKII sequestration may also mimic a naturally occurring process: the self-aggregation of CaMKII holoenzymes into larger clusters under ischemic conditions in neurons.^40^

## CONCLUSION AND PERSPECTIVE

The sophistication of the available tools to study CaMKII is remarkable, and this will certainly enable more progress in the future. However, the most powerful engine to drive new discoveries on CaMKII functions may lie in the availability of three distinct classes of pharmacological inhibitors. These inhibitors now allow a detailed first assessment of CaMKII functions in any given system in a way that is readily accessible to a broad range of scientists without specialized interest in CaMKII research. We hope that this review will facilitate initial discovery through classic pharmacology as well as subsequent validation by complementary methods. We expect that this process will be further enhanced by more detailed additional characterization of the inhibitors and mutants mentioned in this review. This will include filling the gaps in our knowledge about these tools that we have pointed out here, but the most important advances will be in filling the gaps that we do not yet even know about.

## Supplementary Material

1

## Figures and Tables

**Table 1.** CaMKII inhibitors and practical considerations

Drug class	Drug name	Stock solvent (recommended concentration)	Suggested working concentration (μM)	References
CaM-competitive	KN62	DMSO (10 mM)	10	Tokumitsu et al.^43^
	KN93	DMSO or aq.^a^ (10 mM)	10	Sumi et al.^44^
Peptide	tatCN21	aq.	5^b^	Vest et al.^45^
	tatCN19o	aq.	≥2	Coultrap and Bayer,^46^ Deng et al.^47^
	myrCN27	DMSO (1 mM)	5^c^	Lee et al.^48^
ATP-competitive^d^	AS283	DMSO	10	Tullis et al.^26^
	AS397	DMSO	10	Reyes Gaido et al.^49^
	ruxolitinib	aq.	10	Reyes Gaido et al.^49^

a KN93 is available as a water-soluble phosphate salt that can be used similarly to the DMSO soluble version.

b Higher dose (20 μM) can additionally disrupt GluN2B binding.

c 1 μM previously used successfully,^48^ but CN27 (also termed CaMKIINtide) has the same potency as CN21.

d Additional ATP-competitive inhibitors include AS105, GS-680, rimacalib, NP202, bosutinib, and RA608.

**Table 2.** CaMKII tool mutants

Residue	Native effect	Mutant	Direct effect	Functional effect	References
K42	required for nucleotide binding	M or R	nucleotide-binding incompetent	activity abolished; GluN2B binding impaired	Tullis et al.,^82^ Yamagata et al.^83^
D135	catalytic base for phosphorylation	N	increases activation energy	activity likely abolished	Özden et al.,^17^ Chen et al.^84^
C280, M281, C289	*S*-nitrosylation (of Cys) and/or oxidation	VVV^a^	*S*-nitrosylation and oxidation null	autonomous activity induced by *S*-nitrosylation/oxidation abolished	Coultrap and Bayer,^30^ Rumian et al.^31^
T286	(p) autonomous activity; CaM trapping	A^a^	phospho null	pT286-induced autonomous activity abolished; GluN2B binding reduced	Miller and Kennedy,^10^ Giese et al.^85^
		D or E	phosphomimetic	Ca^2+^-independent GluN2B binding	Miller and Kennedy,^10^ Giese et al.^85^
T305/6	(p) impairs stimulated activity; CaM-binding region	AA or VA^a^	phospho null	always CaM-binding competent	Mukherji and Soderling,^32^ Elgersma et al.^86^
		E or D^a^	phosphomimetic	CaM-binding incompetent; activity abolished	Mukherji and Soderling,^32^ Elgersma et al.^86^

(p) denotes autophosphorylation.

a Knockin mouse line available.
