# Supplementary material for: Studying CaMKII: Tools and standards
Source: Cell Rep. Author manuscript; Available in PMC 2024 May 11. (PMC11088445; doi:10.1016/j.celrep.2024.113982)
Supplement: 1 [file NIHMS1988734-supplement-1.pdf]

Cell Reports, Volume *43*

Supplemental information

Studying CaMKII: Tools and standards

Carolyn Nicole Brown and Karl Ulrich Bayer

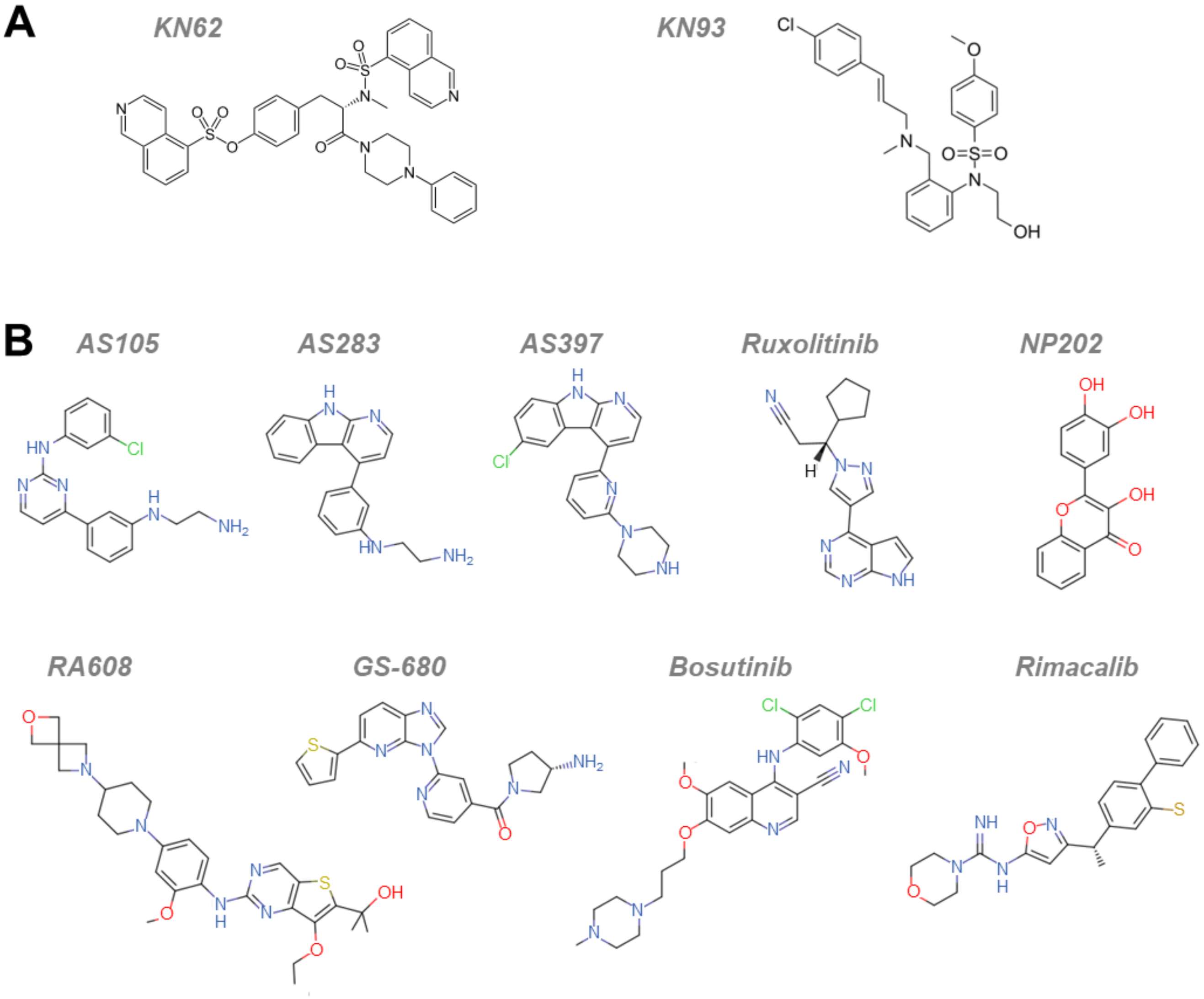

C

AIP	KKALRRQEAVDAL	*CN27*	KRPPKLGQIGRAKRVVIEDDRIDDVLK
AIP2	KKKLRRQEAFDAL	*CN21*	KRPPKLGQIGRSKRVVIEDDR
AC3-I	KKALHRQEAVDAL	*CN19o*	KRAPKLGQIGRQKAVDIED

Supplemental Figure S1. Structures or sequences of CaMKII inhibitors.
(A) Structures of the CaM-competitive CaMKII inhibitors KN62 and KN93.
(B) Structures of various ATP-competitive CaMKII inhibitors.
(C) Amino acid sequences of peptide inhibitors of CaMKII. The inhibitors in the left column are related to the substrate peptide AC2 (KKALRRQETVDAL). Note that the peptide inhibitors by themselves are not cell-penetrating. Cell penetration has been achieved by myristoylation or by N-terminal fusion of the tat sequence (YGRKKRRQRRR).
